# Supplementary material for: Chimpanzee play sequences are structured hierarchically as games
Source: PeerJ. 2022 Nov 16;10:e14294. doi: 10.7717/peerj.14294 (PMC9675342; doi:10.7717/peerj.14294)
Supplement: Supplemental Information 1 — All descriptions and conventions for video coding [file peerj-10-14294-s001.docx]

**Supplementary Material:**

**Chimpanzee play sequences are structured hierarchically as games**

Mielke, Alexander^1,2^ & Carvalho, Susana ^1,3^

*1 Primate Models for Behavioural Evolution Lab, School of Anthropology and Museum Ethnography, University of Oxford, UK*

*2 School of Psychology and Neuroscience, University of St Andrews, UK*

*3 Interdisciplinary Centre for Archaeology and Evolution of Human Behaviour (ICArEHB), Universidade do Algarve, Faro, Portugal*

## Glossary

- Bout: Time between the first play element and the moment all players stop for more than 5 seconds
- Break: Player sits, lies, stands, or walks without displaying any play elements or play face; either watching other players, resting, or performing non-play actions; Break starts when individual stops moving, e.g. after a retreat or chase
- Breakdown: One player screams, flees, or shows aggression
- End: No player performs any play element for more than 5 seconds; time of end is the moment all players go on ‘Break’
- Initiator: First player to perform a play element in a bout or the first new element after a time when all individuals are on break
- Initiation: Any play element by the initiator precedes the first response by any other player or that precedes resumed play after a common break
- Laughter: Inhaled and exhaled panting vocalisation, sometimes called ‘play grunt’
- Play Element: Continuous action in a play context, either directed at a partner, an object, or involving atypical or self-handicapping locomotor behaviours
- Play Face: Open-mouth facial expression; can be full (lower teeth visible) or half (lower teeth covered)
- Sequence: Ordered collection of play elements within one individual
- Transition: Response of one player to the play element of another player
- Turn: Entirety of an individuals play elements between breaks

## Play vs Aggression

Only code Play if there are elements that are unmistakeably non-threatening. For example, if the sequence consists only of one individual approaching and waving an object, and the other one parrying, retreating or retaliating, do not code as play. If there is a very clear play face from one individual, code, but mark ‘aggression’ when play ends without partner responding.

## Ethogram

Abbreviations: F = Focal, P = Partner, O = Object

Format: **Bold** = Extended (theoretically, element could be performed infinitely); *Italics* = short (element has natural start and end, can be counted)

### Contact Play

| **Play Element** | **Description** | **Minimum Action Unit** | **Replacement if rare** |
| --- | --- | --- | --- |
| **Airplane** | F lies on back and throws P in air repeatedly | F lies on back and throws P in air repeatedly | Combine into Move_other |
| *Bite* | F puts body part of P in their mouth | Body contact with mouth open | - |
| **Carry** | F moves while holding other | Support full body weight of P while moving | Combine into Move_other |
| **Drag** | F pulls P along the floor | Moving, holding P, moving their body | Combine into Move_other; Pull |
| *Drum* | F hits P repeatedly with hand and/or feet | Repeated, rhythmic short-term body contact with flat hand | Hit |
| **Embrace** | F puts arm(s) around P | One or both arms press P against F | Mount |
| **Finger in mouth** | F puts finger in mouth of other | Finger extended for P to bite | Touch |
| *Flail_contact* | F throws arms around in flailing motion, hits other because they are too close | Both arms moved around; partner gets hits because they are within reach of the motion, but not one directed hand movement | Hit |
| **Grabble/Wrestle** | F and P hold onto each other and push and pull | Extended grabbing of hair of body part by both participants | - |
| *Head butt* | F hits P with their own head | Fast body contact of head | Hit |
| *Hit* | F makes rapid contact with P using one or both hands | Short-term contact with hand | - |
| **Hold** | F makes continuous contact with P using one or both hands | Long-term contact with hand(s) or feet that prevents movement | - |
| *Jump on* | F jumps on P | Explosive movement, contact with partner body | Step_on |
| *Kick* | F makes rapid contact with P using one or both feet | Fast body contact with feet | - |
| **Mount** | F climbs onto P | Body weight supported by P | Press-down |
| **Parry** | F protects their body from hits by P | F folds or wraps arms around head or other body part to prevent hits | Arm_protect |
| *Pull* | F pulls P towards themselves | Holding hair or body, short-term movement towards F | - |
| *Push* | F pushes P away from themselves | Force exerted as if to displace P, does not have to be effective | - |
| **Press down** | F puts body weight on P to press them to the ground | Extended body contact with downward movement | - |
| **Roll partner** | F rolls the body of the P on the ground while P somersaults | Initiate or support P somersault | Combine into Move_other |
| **Rump rub** | F rubs their genitals and/or rear on P | Genito-genital or rump-rump contact | Mount |
| *Shake partner* | F shakes P’s body back and forth | Repeated back-and-forth movement of holding limp | Combine into Move_other |
| *Shake off* | F shakes own body to remove hold of P | Movement to escape holding | Parry |
| *Step on* | F walks onto P | Short-term contact of lying P | Jump_on |
| **Tickle** | F tickles P | Repeated contact with vulnerable body part | Touch |
| *Touch* | F makes slight contact with P using one or both hands | Short-term careful body contact | - |
| *Trip* | F impedes walking of P by briefly holding or kicking their leg | Short-term contact, P falls | Pull |

### Noncontact Play

| **Play Element** | **Description** | **Minimum Action Unit** | **Replacement if rare** |
| --- | --- | --- | --- |
| **Approach** | F moves towards P | Walks towards P who is not moving; if running, score *Charge*; if other is moving, score *Follow other* (walking) or *Chase* (running); starts the moment they start moving | - |
| **Arm Protect** | F holds arm in front of body as if to block a blow | Arm is held still in front of belly or chest, often while retreating; like ‘Parry’ but without contact | Parry |
| *Arm wave* | F moves arm back and forth above shoulder level | Arm(s) is above shoulder level at some point during movement, flailing around; mark new movement every time arm is returned to position in front of body | Arm_swing |
| *Arm raise* | F moves one arm or both arms above their head | Arm(s) raised above head level, no flailing; mark new movement every time arm is returned to position in front of body | - |
| *Arm swing* | F moves one or both arms before its body back and forth | Arm moved from one side to other in front of body; mark new movement every time arm is returned to position in front of body | - |
| *Beckon* | F moves hand in a sweep from the elbow or a twist from the wrist towards P | ‘Come hither’ gesture | Reach |
| **Bend Sapling** | F bends little tree or sapling to get access to partner or facilitate play | F has at least one hand on tree, bends it | Combine into Move_substrate |
| **Bipedal** | F stands up on hindlegs | F arms leave the ground and they are straight and hold the position; if they only go up quick, use *Bop*; if they move backwards or forwards, use *Swagger*; does not have to be marked for every single action after they went bipedal, only for the moment they move up | - |
| **Bop** | F jumps up and down repeatedly in the same spot | Bouncing up and down, sometimes mixed with *Bow*; use if hands leave ground briefly | Swagger |
| *Bow* | F quickly bends arms and/or legs while standing | Front goes down more than backside; if it is held, mark *Head down* or *Look between legs* | Crouch |
| **Branch pull** | F pulls substrate that the P is hanging from | P has to be on substrate, otherwise it’s B*ranch shake* | Combine into Move_substrate |
| **Branch shake** | F shakes branch back and forth | No partner on branch, can also be a small tree | Combine into Move_substrate |
| *Charge* | F runs towards P in burst | F does not run past spot where P is initially, otherwise it is *Chase*; if F walks, it’s approach | Approach |
| **Chase** | F runs after P who is running away | Both individuals have to run and pass initial spot of P | Follow-other |
| **Circle tree** | F runs or walks around structure in a circle | Paired with *Lead* or *Follow other* to denote the role in the circling | Follow-other |
| **Circle partner** | F runs or walks around P in a circle | P has to somehow hold still | Pass |
| **Climb** | F climbs up or down substrate | Just note the beginning, don’t need full information on what happened in tree or ground; it’s Climb if all limps leave the ground | - |
| **Crouch** | F is quadrupedal and flexes limbs for extended period | Belly on the ground, hind and front legs bent, not *Rocking* or *Bowing* | Bow |
| **Display** | F performs exaggerated display of physical prowess | This can include a number of other elements that should also be marked; so, can be *dragging object*, *swagger*, *throw* things, go piloerect, *shake branch*, *approach* other etc | Swagger |
| *Drum_tree* | F hits a tree with their hand | One hit is enough; not used if they just hold onto tree for other reasons; can be combined with *Kick_tree* if they do both | - |
| *Fall* | F was hanging from substrate and falls off | Does not have to be deep, just indicates that they are back on the ground | Jump |
| *Feint* | F rapidly changes direction of movement | Running, walking or retreat; suddenly change of direction, often accompanied by *Bow* | - |
| *Flail* | F makes hitting or throwing gestures without O or hitting P | No contact to partner and no object involved, but scary arm movement, often bipedal or while swaggering, usually both arms, often with bent arms | Hit_attempt |
| **Flee** | F runs away from P fast | Running (otherwise *Retreat*). Will sometimes over exaggerate the movements and play face at P to make sure it is play | Retreat |
| **Follow Other** | F slowly walks after other individual | Like approach, but other individual is moving as well; Both individuals are walking or circling tree; one leads, one follows; does not have to be marked if other *Retreats, Retreats backwards,* or *Flees;* does not have to be marked when *Chase* is marked | - |
| *Hand fling* | F makes rapid movement of the hand or arm in the direction of the recipient | Arm is mostly stable, only hand is moved | Arm-wave |
| **Hang** | F is suspended from substrate or P | Hanging from tree while still engaged in play; sometimes mixed with *Rock* (if individuals hangs in one place but wiggles), *Head* *down* (if they hang head first); not marked when *Swing* is marked | Swing |
| **Head down** | F stands with head down, rear up | Like a very deep *Bow* that is held over time. Weight not on head (as in *Headstand*); does not have to be used if *Look between legs* is used | - |
| *Head Scratch* | F holds arm over head and scratches the head at least once | Scratches part of the head; often seen during arm raise, on the way of the arm down | Loud_scratch |
| *Head shake* | F shakes head back and forth or nods up and down | Sometimes hard to see, often while approaching other or running away | - |
| **Head stand** | F bends forward and places head on the ground | Weight rests at least partially on head | Head_down |
| **Hide** | F moves behind tree or other obstacle to avoid P | While retreat from other, bringing obstacle between F and P (even if only briefly); not *Circling* the object | - |
| *Hide_swing* | F holds onto tree and swings around it | Not *Circling* because they don’t make a whole revolution; if part of fleeing, mark fleeing before and after | Hide/Swing |
| *Hit_attempt* | F makes directed hitting movement with one hand but misses | Different from *Flail* in that movement is directed at body and not just arms being thrown around in from of body; one-handed | Flail |
| **Ignore** | F does not react to Ps play elements and continuous previous activity | Just sits there watching a perfectly fine invitation | NA |
| *Kick dirt* | F kicks sand, leaves, or soil with their feet | Like *Rake ground*, but with feet | Rake_ground |
| *Kick_tree* | F kicks tree | Contact with tree that is not just part of swinging around tree (*Hide_swing*) | Drum_tree |
| **Lead** | F walks ahead of P | Both individuals are walking exaggeratedly or circling tree; one leads, one follows; does not have to be marked if other *Retreats, Retreats backwards,* or *Flees;* | Retreat |
| **Lie down** | F lies on their back or side | As part of the play, not as end of the play to do something better | - |
| **Look between legs** | F bends over and looks at partner through legs | Like *Head down*, but clearly making eye contact with P through legs | Head_down |
| *Loud scratch* | F makes loud exaggerated scratching movement on own body | Not just boredom or itch; big movements | - |
| *Pass* | F walks by other individual in very close distance or between their legs | Deliberately brushing other as invitation to follow | Approach |
| *Pirouette* | F turns around their vertical axis | Do not mark *bipedal* or *arm raise* if they are part of the pirouette; does not have to be 360 Degrees if individual is hanging off a branch | Somersault |
| *Present body part* | F moves body to deliberately expose an area to the recipients attention | Do not mark when the *Look between legs* | - |
| *Press ground* | F puts weight on one or both hands | Often accompanied by little B*ow* or following *Bop* | Slap_ground |
| **Pretend Throw** | F makes throwing movement above their head without holding an O | Arm held above head and then explodes forward, no object | Flail |
| **Rake ground** | F pulls leaves or sand around | Fingers enter sand or leaves, makes grooves in underground | Kick_dirt |
| **Retreat** | F walks or jumps away from P while facing away from P | Not running, not facing P, moving forwards away from P | - |
| **Retreat backwards** | F walks or jumps away from P while facing P | Not running, moving backwards away from P; do not mark if they just take a backwards step to do other action | Retreat |
| *Reach* | F extends arm to recipient with hand in an open, palm upwards position | Looks like begging; arm held out, palm upwards | - |
| **Rock** | F moves side-to-side or back and forth repeatedly | Sitting or standing in one place, more than one change in balance; if only one change in balance, put *Press_ground* (if they balance to the front) or *Bop* (if they balance back) | - |
| **Run-stiff** | F moves fast and exaggerated | Moving away from or towards P, fast | Approach |
| **Run parallel** | F and P run next to each other in the same direction | Both run, not towards or away from each other | Follow_other |
| *Slap ground* | F hits the ground with one or both palms | Palm makes contact with ground; if they come back down after bopping or being bipedal, that is not a slap of the ground; use *Press ground* if they use the knuckles or movement is not rapid | Press_ground |
| *Somersault* | F rolls around its horizontal axis | Complete rotation around body axis; otherwise could be headstand or bow | Pirouette |
| *Stomp* | F lifts foot is lifted vertically and brings it into contact with ground | Has to be different from just stepping down during normal movement | Press_ground |
| **Swagger** | F walks bipedally while swinging arms on the side | Looks like a mix of bipedal and flailing, often combined with piloerect | Bipedal |
| **Swing** | F swings back and forth or brachiates while hanging from substrate | Moving while hanging from substrate | Hang |
| **Stare at** | F looks intently at partner | Only mark if F does not do anything else or makes clear effort to get Ps attention by making eye contact; like peering | - |
| **Walk (stiff)** | F walks quadrupedal with a slow exaggerated movement | Moving shoulders while walking, staring at partner, bouncing around | Run_stiff |

### Social Object Play

| **Play Element** | **Description** | **Minimum Action Unit** | **Replacement if rare** |
| --- | --- | --- | --- |
| Beg_object | P has O, F extends hand with palm up to receive object | Hand extended, no direct contact with object (which would be *Grab*) | Combine into Explore_object |
| Bite_object | F bites O | O in mouth; no matter whether pressure applied | Combine into Explore_object |
| Break_object | F breaks O or removes O from substrate | O removed from substrate or broken in multiple pieces | Combine into Explore_object |
| Carry_object | F moves while holding O in hands, feet, or mouth | O lifted off ground, F moves | Combine into Explore_object |
| Circle_object | F runs or walks around O | O stationary, F moves around | Circle_tree |
| Cover face | F covers face with O | O used to cover eyes, as if mask | Combine into Explore_object |
| Drag_object | F pulls O along the floor | O moved while contact with floor | Combine into Explore_object |
| Drop_object | F drops O | O leaves hand of F | Combine into Explore_object |
| Drum_object | F hits O repeatedly | Repeated contact with hands or feet | Drum_tree |
| Explore_object | F manipulates O while holding it | F holds O, looks intensely, sniffs etc | Combine into Explore_object |
| Grab_object | F takes O or substrate that P is on | F picks up O or keeps hand/foot on it but does not move around | Combine into Explore_object |
| Head flagging | F repeatedly looks between O and P | At least 2 iterations, O not moving but F face goes back and forth | Combine into Explore_object |
| Hit_object | F hits other individual or substrate with O | Non-repetitive contact of O with either hands or other object | Drum_tree |
| Jump on_object | F jumps on top of O | O on ground, F jumps and lands on it with feet | Combine into Explore_object |
| Kick_object | F hits O with feet | Like hit, but contact with feet | Kick_tree |
| Poke_object | F pokes P or other O with O | No hitting, but exploratory contact of O with stick | Explore_object |
| Present_object | F puts forward O for P to explore | F in possession of O; changes position of O to facilitate contact between F and O | Combine into Explore_object |
| Pretend-throw_object | F makes throwing movements with O but does not let go | Can be overhead or underhand throw; differs from waving because it is aimed at partner | Combine into Explore_object |
| Pull_object | F pulls O towards themselves | F not moving position, but moves position of O closer to themselves | Combine into Explore_object |
| Push_object | F pushes O away from themselves | F not moving position, but moves position of O away from themselves | Combine into Explore_object |
| Shake_object | F shakes O | Repeated movement of O in different directions | Combine into Explore_object |
| Slap_ground_object | F makes contact between O and ground | O is not dropped or thrown, but actively smashed on the ground without letting go | Slap_ground |
| Take_object | F takes O from P | P is possessor before; F becomes possessor afterwards | Combine into Explore_object |
| Throw_object | F throws O away | Change of position of O with some airtime | Combine into Explore_object |
| Tickle_object | F tickles P or self with O | O used on body of P or F leads to tickling response | Tickle |
| Touch_object | F gives slight touch to O | Short light contact, then remove | Combine into Explore_object |
| Tug of war | F and P hold O, pull in different directions | Two individuals simultaneously hold O, try to move in different direction | - |
| Twist object | F holds O with P and they twist it together | Both individuals have contact to object, twisting motion by at least one individual | Combine into Explore_object |
| Wash_object | F puts O in water or cleans it with their hands | O in water | Combine into Explore_object |
| Watch_object | F watches P handle O | P does something with object, F peers | Combine into Explore_object |
| Wave_object | F waves O in direction of P threateningly | Up and down or back and forth movement with object, looking at P | Flail |
| Wrap_object | F wraps vine or fruit around body part | Soft O, put around body | Combine into Explore_object |

### Non-Play: can look similar to play elements, but not part of play interaction

| **Behaviour** | **Description** |
| --- | --- |
| Groom | F grooms with P |
| Aggression | Contact or noncontact aggression against player or non-player |
| Copulation | F copulates with P |
| Embrace | F puts one or both arms around P |
| Touch | F touches body of P or non-player |
| Reassurance | F either gives or seeks physical contact after aggressive interaction |
| Supplant | F takes over other individuals resource |
| Arm Reach | F reaches out in a begging gesture |
| Arm Wave | F angrily waves arm at other |
| Bark | Barking vocalisation |
| Drum | Drumming ground or tree |
| Fear Grin | Bared teeth facial expression |
| Grunt | Grunting vocalisation |
| Hoo | Hoo vocalisation |
| Leaf Clip | F rips leaf apart with their mouth |
| Lip Smack | Lip smacking vocalisation |
| Loud Scratch | Big loud scratching of body part |
| Pant Hoot | Pant hoot vocalisation |
| Pant Grunt | Pant grunt vocalisation |
| Present | F moves body to deliberately expose an area to the recipients attention |
| Scream | Screaming vocalisation |
| Shake Branch | F shakes branch up and down or back and forth at receiver |
| Slap Ground | F uses one or both hands to slap the ground with their palms |
| Tap Foot | F repeatedly taps ground or O with foot |
| Whimper | Whimpering vocalisation |

### Possible Outcomes of Play

| **Outcome** | **Description** |
| --- | --- |
| Ignore | One player stops reacting to the play |
| Flee | One player runs away from play bout |
| Aggression | One or both players start aggression |
| Other Interaction | Players move from playing to some other interaction type |
| Unknown | It is not clear why play stopped |

### Video-level Information

- Time
- Date
- Play included
- Party Composition

## Coding Conventions

- Only code play face if it is very clearly visible
- For repeated actions, code every step (e.g., when drumming, mark every time the hand hits the object or partner; if individual hangs off branch and bows and then jumps repeatedly, mark ‘Hang-Bow-Jump’ as often as necessary)
- Objects that lie on the ground are considered ground until they are manipulated

## Contentious Codes

If there are multiple possible codes, check here as to which one is appropriate

**Focal moving towards partner**:

- Approach: Focal walks, partner is still
- Follow: Focal walks, partner walks
- Charge: Focal runs, partner is still
- Chase: Focal runs, partner runs
- Run parallel: Focal runs, partner runs, next to each other

**Focal moves away from partner**

- Flee: Focal runs
- Retreat: Focal walks away facing away
- Retreat backwards: Focal walks away facing towards partner
- Lead: Focal walks away slowly with partner following, often while contact is maintained

**Focal rears up on two legs**

- Bop: Focal’s hands briefly leave the ground but return almost immediately
- Bipedal: Focal rears up on legs with arms leaving the ground and stays that way
- Swagger: Focal moves towards or away from the partner on legs only

**Focal flexes arms**

- Bow: Focal moves only arms down, often just briefly
- Bop: Focal moves up and down
- Crouch: Focal flexes both arms and legs, often holds the position
- Head down: Legs are straight, arms are flexed so that the head is below the pelvis
- Headstand: Weight put on head
- Looking through legs: Actively make eye contact with partner through legs

**Focal makes arm movements**

- Arm Swing: arm is moved to the side, in front of body
- Flail: Focal makes hitting motions with both arms without hitting partner
- Arm Raise: Focal moves arm(s) straight up and takes them straight down
- Pretend throw: Focal moves arm(s) straight up but then makes throwing movement forward
- Arm Wave: Focal moves arms up but then waves then around there
- Reach: Focal moves arm straight to the front and holds hand out
- Hand fling: Focal moves arm to front and flings hand towards partner
- Hit attempt: Focal makes hitting movement with one hand that is intended to hit but misses
- Parry: Partner makes contact, focal moves arms around to ward of hit

**Focal makes contact with hands**

- Touch: Light contact, non-threatening
- Hit: Hard short contact with palm, fist, or arm
- Push: Short or long contact with palm or arm that moves partner away from the focal
- Flail_contact: One or both arms moved around non-directly in front and above body, contact incidental and sometimes with both hands
- Hold: Long contact with palms or hands that anchors partner in space
- Parry: Partner hits or flails, use arms or hands to stop partner movement
- Hold/ Mount/ Swing: Holding onto other and swinging from their back while they turn

**Focal moves object around in the air**

- Wave object: Individual moves object above the head or to the side and moves it back and forth without hitting anyone
- Hit with object: Individual moves object towards partner and actually makes contact with the object
- Pretend throw: Focal holds object above head and makes rapid movement towards partner, or holds it underhand and makes rapid movement towards partner, but does not let go of object
- Throw object: Focal actually lets go of the object
- Slap ground object: individual takes object and bangs it on the ground with force
- Flail (with object coded): Individual moves one arm around and holds an object in the other hand

**Focal makes deliberate contact with the ground**

- Slap ground: Palm down, rapid movement
- Press ground: Palms or knuckles, slow movement, often rather change of balance onto hands
- Bop: Pressing ground to move up and land, but no additional pressure

**Focal climbs tree, swings on tree, and hits partner either with branch or by landing on them**

- Swing/Branch shake: branch hits partner, focal does not touch the partner
- Swing/Branch shake/Jump on: branch hits partner, focal touches the partner and stays on partner
- Swing/ Jump on: branch does not hit partner, focal touches the partner and stays on partner
- Swing/ Kick: branch does not hit partner, focal touches partner briefly

**Focal holds onto part of the substrate**

- Branch shake: substrate is only a branch of a larger tree, no partner on the branch
- Branch pull: substrate is only a branch of a larger tree, partner is on the branch
- Bend sapling: substrate is a small tree or sapling, partner is not on it
- Branch pull/ Bend sapling: pull sapling partner is on
- Bend sapling/ Branch shake: shake sapling at partner to hit them
